# Steroid replacement in primary adrenal failure does not appear to affect circulating adipokines

**DOI:** 10.1007/s12020-014-0388-6

**Published:** 2014-08-17

**Authors:** Marta Fichna, Piotr Fichna, Maria Gryczyńska, Agata Czarnywojtek, Magdalena Żurawek, Marek Ruchała

**Affiliations:** 1 Institute of Human Genetics, Polish Academy of Sciences, 32 Strzeszynska, 60-479 Poznan, Poland; 2 Department of Endocrinology and Metabolism, Poznan University of Medical Sciences, 49 Przybyszewskiego, 60-355 Poznan, Poland; 3 Department of Paediatric Diabetes and Obesity, Poznan University of Medical Sciences, 27/33 Szpitalna, 60-572 Poznan, Poland

**Keywords:** Addison’s disease, Cortisol, Leptin, Adiponectin, Resistin

## Abstract

Despite continuous efforts for an optimal steroid replacement, recent observations suggest increased cardiometabolic risk and related mortality in primary adrenal insufficiency (PAI). Adipokines are peptides from the adipose tissue, markers of cardiometabolic dysfunction. This study was aimed to evaluate serum levels of adipokines: leptin, adiponectin, and resistin in PAI during conventional steroid substitution. The analysis comprised 63 patients (mean age 42.7 ± 14.1 years) and 63 healthy controls. Serum adipokines, lipid profile, and plasma glucose were assessed in both cohorts. ACTH, serum insulin, HOMA-IR, DHEA-S, cortisol and 24 h urinary free cortisol were determined in PAI. Body mass composition was analyzed by Dual-Energy X-ray Absorptiometry. Mean BMI in the control group was 24.1 ± 3.9 kg/m^2^ and 23.7 ± 3.9 kg/m^2^ in the PAI cohort. Serum leptin and adiponectin levels were similar in both groups, whereas resistin appeared significantly lower among affected subjects (*p* = 0.0002). Its levels were weakly correlated with HOMA-IR (*p* = 0.048). Leptin was independently correlated with fasting insulin, HOMA-IR, BMI, and body fat (*p* < 0.001). At the multiple regression analysis only weight (*p* = 0.017), total and HDL cholesterol (*p* < 0.001) appeared significant predictors of adiponectin level. No adipokine correlations with serum cortisol or daily hydrocortisone dose were found. Patients receiving DHEA substitution displayed lower leptin and adiponectin levels (*p* < 0.05). In conclusion, our study did not provide evidence of an adverse adipokine profile in patients with PAI under conventional glucocorticoid replacement. Serum adipokines in treated PAI follow similar correlations to those reported in healthy subjects. Further prospective studies are warranted to verify and explain plausible excess of cardiovascular mortality in PAI.

## Introduction

Primary adrenal insufficiency (PAI), also known as Addison’s disease, is traditionally accounted among rare disorders. Nonetheless, recent epidemiological data from the developed countries indicate that its prevalence may exceed 100 cases per million, and a considerable rise in autoimmune etiology is currently noted [1]. PAI treatment relies upon exogenous steroid substitution; however, standard oral replacement schemes fail to perfectly mimic the natural circadian rhythm of cortisol secretion [2]. Optimization of the individual hormone dosage and long-term monitoring remain major clinical challenges. In case of insufficient glucocorticoid substitution patients are threatened by potentially lethal adrenal crisis, while chronic over-replacement may lead to adverse effects, such as glucose intolerance, enhanced visceral adiposity, and bone demineralisation [3]. Former reports substantiated normal life expectancy in properly diagnosed subjects with PAI [4, 5], while recently published observations suggest that increased death rates persist for many years after diagnosis [6–8]. Excess mortality in PAI is due to infection, trauma and other common causes of the adrenal crisis [6] but unexpectedly might also be connected with cardiovascular diseases and malignancies [7, 8]. Although no unequivocal evidence of increased cardiovascular morbidity was demonstrated to date, elevated incidence of cardiometabolic risk factors, including abdominal obesity, dyslipidemia, and impaired glucose tolerance was reported in PAI [3, 9, 10]. These features, hallmarks of the metabolic syndrome, might possibly be accounted for by superfluous glucocorticoid treatment.

Recent studies reveal that altered serum levels of adipokines, such as leptin, adiponectin, and resistin, may reflect increased cardiometabolic risk [11–14]. Adipokines are biologically active peptides, which originate from the adipose tissue. In the auto-, para- and endocrine manner, they affect and integrate the energetic balance, food intake, glucose and lipid metabolism. Leptin was one of the first known adipose tissue hormones [15]. It acts as central lipostatic signal, limiting the food ingestion and increasing the energy expenditure [16, 17]. Leptin is mainly synthesized by the subcutaneous adipose tissue and its plasma levels increase with the weight gain, suggesting that common human obesity is a leptin-resistant state [15]. Apart from overfeeding, leptin secretion is stimulated by insulin, glucocorticoids, TNFα, and estrogens [18]. Leptin replacement in patients homozygous for its gene mutations and in subjects with lipodystrophy syndromes who exhibit reduced leptin levels, results in beneficial effects on body mass composition, improved lipid profile and glucose metabolism [19].

Adiponectin is exclusively produced by adipocytes, especially in subcutaneous fat, but in contrast to leptin, its synthesis declines in obese subjects [20]. Adiponectin release in vitro is triggered by insulin and PPARγ agonists, and suppressed by TNFα, glucocorticoids, and androgens [21]. Its receptors are mainly located in muscle and liver, where adiponectin stimulates oxidation of the fatty acids, inhibits hepatic gluconeogenesis and improves insulin sensitivity [22]. Adiponectin presents anti-inflammatory and anti-atherogenic action, decreasing the synthesis of endothelial adhesion molecules, foam cell generation, and smooth muscle proliferation [11, 22]. Adiponectin deficiency is connected with insulin resistance, impaired glucose tolerance, hyperlipidemia, and high risk of cardiovascular disease [11, 21, 23].

Resistin was named by its ability to induce insulin resistance [24]. Its expression prevails in visceral adipose tissue, and may be stimulated by feeding, glucose, dexamethasone and androgens [25]. In rodent models, resistin impairs insulin action and glucose tolerance [24]. It up-regulates hepatic gluconeogenesis enzymes and reduces the expression of insulin receptor substrate-2, a cytoplasmic adaptor that mediates downstream insulin effects [25, 26]. Presumably resistin might represent a link between obesity, insulin resistance and diabetes. However, metabolic relevance of human resistin remains ambiguous. The protein shares only 60 % amino acid identity with its rodent homologue, its expression seems to prevail in macrophages and is differently regulated [25, 27]. Data correlating circulating resistin with human obesity and insulin resistance are contradictory [28, 29]. Nonetheless, resistin displays adverse vascular effects, comprising increased expression of vascular adhesion molecules and proinflammatory cytokines, together with enhanced smooth muscle proliferation [12].

Broadly, adipokines appear markers of metabolic and cardiovascular pathology and their serum levels may indicate elevated disease risk [11–13, 20, 30]. Therefore, it seemed of interest to investigate circulating adipokines in a population with primary adrenal failure, suspected to be at particular risk for cardiometabolic morbidity and mortality. The current analysis was designed to evaluate serum levels of leptin, adiponectin, and resistin in a series of PAI patients during conventional steroid replacement therapy, and to investigate their correlation with clinical, biochemical and hormonal parameters, which may be connected with cardiometabolic dysfunction.

## Materials and methods

The study comprised 63 patients (41 females and 22 males) with PAI of autoimmune etiology and 63 healthy sex-, age- and BMI-matched control subjects, all issued from Polish population of Caucasian descent. The mean age of affected individuals was 42.7 ± 14.1 years and mean duration of glucocorticoid replacement was 11.0 ± 10.4 years (range 1–45 years). The diagnosis of PAI was based on typical signs and symptoms (weakness and fatigue, hypotension and postural symptoms, anorexia, weight loss, nausea/vomiting, salt-craving, hyperpigmentation of skin and mucosae), basal serum cortisol levels below 83 nmol/l (3 μg/dl), accompanied by plasma ACTH exceeding 22 pmol/l (100 pg/ml), and corroborated by lack of response of the adrenal cortex to intravenous stimulation with synthetic ACTH_1–24_ (peak serum cortisol less than 500 nmol/l = 18 μg/dl) [1]. Autoimmune origin of the disease was confirmed with positive serum autoantibodies to 21-hydroxylase evaluated using RIA assay (RSR Ltd, Cardiff, UK). Daily hydrocortisone (HC) dose ranged between 7.5 and 35 mg/d (mean 24.1 ± 5.8 mg/d), and for the purpose of the study was corrected for the body weight in kilograms (mean dosage 0.36 ± 0.10 mg/kg). Forty-one (65 %) patients were on twice daily regimen, and the remaining subjects were taking HC three times per day. The majority of patients (81 %) received mineralocorticoid replacement with daily fludrocortisone (0.05–0.1 mg) to normalize blood pressure (BP) and serum electrolytes. Additionally 19 of them have been treated with 10–25 mg of dehydroepiandrosterone (DHEA) per day for at least 6 months before the analysis. At the time of the study all patients were either euthyroid or on stable levothyroxine replacement conferring TSH and thyroid hormone levels within the reference range. Individuals with known coexisting diabetes were excluded. Patients were enrolled from the inpatient and outpatient endocrine clinics at Poznań University of Medical Sciences. The control group was recruited among blood donors from the Regional Blood Transfusion Centre in Poznań. Their mean age was 42.8 ± 12.7 years. None of them was taking any medication interfering with the hypothalamo-pituitary-adrenal axis. Informed consent was obtained from all participants, and the study was approved by the local ethical committee at Poznań University of Medical Sciences.

Clinical evaluation of study participants comprised weight, height, and BP measurements at noon (mean of three independent measurements on consecutive days). Blood samples were drawn in the morning after overnight fast. Serum adipokines were evaluated in all participants using commercially available kits based on radioimmunoassay techniques. Leptin levels were assessed by means of Human Leptin RIA-CT (DIAsource ImmunoAssays S.A., Nivelles, Belgium), with the intra- and interassay coefficients of variation (CV) 4.8 and 5.3 %, respectively. Adiponectin was analyzed with Human Adiponectin RIA Kit (Millipore, MA, USA) featuring the respective intra- and interassay CVs 6.2 and 9.2 %. Serum resistin concentration was evaluated using Resistin (43–65) (Human) RIA Kit (Phoenix Pharmaceuticals, CA, USA), with the detection limit 153.3 pg/ml. Serum DHEA sulfate (DHEA-S), insulin, cortisol and 24 h urinary free cortisol (UFC) excretion were determined in affected subjects by electrochemiluminescent method (ECLIA) using Modular Analytics E170 and commercial kits from Roche Diagnostics. Plasma ACTH was assessed in PAI patients by IRMA (Brahms GmBH, Hennigsdorf, Germany). Other laboratory analyses comprised fasting plasma glucose, triglyceride, total cholesterol and its high-density lipoprotein (HDL) fraction and were performed in all subjects (except for tryglicerides in controls) by standard laboratory methods applied in our tertiary centre. BMI was calculated as weight (kg)/height (m) squared. Low-density lipoprotein (LDL) cholesterol was determined by Friedewald’s formula. Insulin resistance was studied using the homeostasis model assessment of insulin resistance (HOMA-IR) calculated by the formula: fasting serum insulin (μU/ml) × fasting serum glucose (mmol/l)/22.5.

Moreover, body mass composition was analyzed in 39 patients with PAI by Dual-Energy X-ray Absorptiometry method (Lunar DPX; Lunar Corp. Madison, WI).

Statistical analyses were performed by means of GraphPad Prism v.6.0 software (La Jolla, CA, USA). Data are presented as mean ± standard deviation (±SD) unless stated otherwise. Normally distributed data were compared using unpaired Student *t* test, whereas those with non-normal distribution were analyzed by nonparametric Mann–Whitney test. Statistical correlations were assessed by calculation of Pearson’s or Spearman’s coefficient, depending on data distribution. Multivariate linear regression was applied to test for independent relationship of parameters, which displayed significant correlations in univariate model. Two-tailed *p* values <0.05 were considered statistically significant.

## Results

Mean BMI value in the control group was 24.1 ± 3.9 kg/m^2^ and 23.7 ± 3.9 kg/m^2^ in the PAI cohort. Considering the WHO classification of the body mass according to the BMI value, 4 (6.3 %) PAI patients were underweight, 41 (65.2 %) presented normal weight, 14 (22.2 %) were overweight, and 4 (6.3 %) obese. Body mass composition demonstrated mean 29.9 ± 11.2 % body fat proportion and 28.4 ± 11.2 % trunk fat content among PAI patients. Blood pressure measurements revealed mean systolic BP 118 ± 13 mmHg and mean diastolic BP 76 ± 7 mmHg in the studied PAI group. As expected, serum cortisol levels in healthy subjects were considerably higher than in PAI patients (417.8 ± 169.1 vs. 36.5 ± 42.5; *p* < 0.001). Only limited biochemical analyses were performed in the control group; however, no significant differences were found for the fasting glucose and cholesterol fractions compared to the PAI cohort (Table 1).

**Table 1:** Biochemical and hormonal findings in patients with primary adrenal insufficiency (PAI) and healthy controls

Unit	PAI patients (*n* = 63)		Healthy controls (*n* = 63)		*p* value
	Mean ± SD	Range	Mean ± SD	Range	
Fasting glucose (mmol/l)	4.7 ± 0.7	3.4–6.2	4.8 ± 0.7	3.2–6.8	0.257
Fasting insulin (mU/l)	8.8 ± 6.6	2.6–32.6	NA		
HOMA-IR	1.9 ± 1.7	0.4–7.6	NA		
Total cholesterol (mmol/l)	5.4 ± 1.3	2.8–9.0	5.2 ± 1.1	2.9–8.1	0.601
HDL (mmol/l)	1.7 ± 0.5	0.9–3.1	1.6 ± 0.5	0.9–2.8	0.457
LDL (mmol/l)	3.0 ± 1.1	0.9–6.0	2.9 ± 1.0	1.1–5.3	0.582
Triglycerides (mmol/l)	1.4 ± 0.7	0.4–3.9	NA		
ACTH (pg/ml)	434 ± 444	18–1569	NA		
Cortisol (nmol/l)	36.5 ± 42.5	0–163.6	417.8 ± 169.1	155.3–721.5	**<0.001**
DHEA-S (μg/dl)	64.4 ± 97.9	0.1–209.6	NA		
UFC (nmol/24 h/m^2^)	197 ± 101	43–403	NA		

Statistically significant value is given in bold

*NA* non-assessed, *UFC* urinary free cortisol

**Fig. 1:**
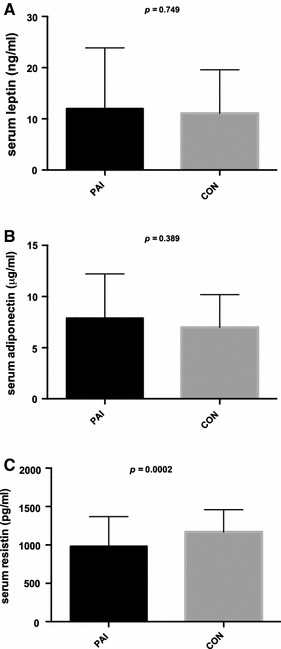
Serum adipokine levels in patients with primary adrenal insufficiency (PAI) and healthy control subjects (CON): **a** serum leptin, **b** serum adiponectin, **c** serum resistin

Serum adipokine evaluation revealed that leptin levels in PAI cohort were comparable to healthy controls (12.0 ± 11.8 vs. 11.1 ± 8.5 ng/ml; *p* = 0.749) (Fig. 1a) and higher in females compared to males (*p* < 0.001 in both healthy and affected subjects). Leptin concentration in PAI did not correlate with age and serum cortisol (*p* > 0.05) although these correlations were present in the control group (*r* = 0.43, *p* < 0.001 and *r* = 0.34, *p* = 0.016, respectively). In PAI cohort leptin was correlated with serum DHEA-S (*r* = −0.47, *p* < 0.001), HDL cholesterol (*r* = 0.34, *p* = 0.006), fasting insulin (*r* = 0.57, *p* < 0.001) and HOMA-IR (*r* = 0.56, *p* < 0.001). It also revealed positive correlation with BMI (*r* = 0.26, *p* = 0.04) and with fat proportion in whole body (*r* = 0.74, *p* < 0.001) and trunk (*r* = 0.68, *p* < 0.001). No correlation between leptinemia and disease duration, daily HC dose, 24 h urinary free cortisol, ACTH, total cholesterol and triglycerides was detected. Parameters displaying significant correlation with leptin level were tested in multivariate analyses for independent associations. Circulating leptin in PAI appeared independently correlated with fasting insulin and HOMA-IR, BMI, and the proportion of total and trunk body fat (all *p* < 0.001). Correlation analysis in control subjects confirmed well-known relationship between leptin and BMI (*r* = 0.31, *p* = 0.009) but did not provide evidence for a link between leptin and cholesterol profile (*p* > 0.05).

Circulating adiponectin levels in PAI subjects were also similar to controls (7.90 ± 4.31 vs. 6.88 ± 3.28 μg/ml, *p* = 0.389) (Fig. 1b). In both groups adiponectin was significantly higher among females than in males (*p* < 0.001 in PAI and *p* = 0.002 in controls). There were negative adiponectin correlations with weight (*r* = −0.30, *p* = 0.019), BMI (*r* = −0.23, *p* = 0.025), HOMA-IR (*r* = −0.352, *p* = 0.002) and serum DHEA-S (*r* = −0.33, *p* = 0.009) among PAI subjects. Positive correlations with total cholesterol (*r* = 0.44, *p* < 0.001) and its HDL fraction (*r* = 0.50, *p* < 0.001) were also found. Only a borderline negative correlation with the proportion of whole body fat (*r* = −0.14, *p* = 0.056) was detected, whereas no relationship with the trunk fat, PAI duration, daily HC dose, plasma ACTH, serum cortisol, LDL cholesterol, triglyceride concentration or 24 h UFC excretion was discovered (*p* > 0.05). At multiple regression analysis only weight (*p* = 0.017), total and HDL cholesterol (*p* = 0.0025 and *p* = 0.0003, respectively) remained significant predictors of adiponectinemia in PAI. Similar adiponectin correlations appeared significant in the control group: weight (*r* = −0.33, *p* = 0.007), BMI (*r* = −0.29, *p* = 0.012), and HDL cholesterol (*r* = 0.43, *p* < 0.001).

Serum resistin was significantly decreased in PAI patients compared to healthy controls (982 ± 386 vs. 1169 ± 286 pg/ml, *p* = 0.0002) (Fig. 1c) and did not differ between females and males. Resistin levels in PAI were weakly correlated with HOMA-IR (*r* = 0.14, *p* = 0.048) and independent of athropometric, other biochemical and hormonal parameters (*p* > 0.05). Likewise, no statistically significant correlation was found between circulating resistin and BMI, fasting glucose or cholesterol profile in controls (*p* > 0.05).

Predictably, patients receiving DHEA replacement displayed significantly higher serum DHEA-S levels compared to those without supplementation of adrenal androgens (Table 2). These circumstances allowed evaluating the effect of androgens on adipokine levels. In line with the correlation analyses, patients on DHEA treatment presented significantly lower leptin (*p* = 0.040) and adiponectin (*p* = 0.017) levels compared to those without supplementation of adrenal androgens (Table 2). On the contrary, no effect of DHEA on circulating resistin was noted (*p* = 0.664).

**Table 2:** Serum dehydroepiandrosterone sulfate (DHEA-S) and adipokine levels in patients with primary adrenal insufficiency receiving dehydroepiandrosterone replacement (on DHEA) and those without DHEA treatment (no DHEA)

Unit	On DHEA (*n* = 19)		No DHEA (*n* = 44)		*p* value
	Mean ± SD	Range	Mean ± SD	Range	
DHEA-S (μg/dl)	221.7 ± 133.9	77.2–503.0	13.5 ± 20.2	0.1–74.7	**<0.001**
Leptin (ng/ml)	8.3 ± 9.9	0.3–43.2	17.1 ± 16.6	0–60.1	**0.040**
Adiponectin (μg/ml)	6.1 ± 3.8	0.8–15.6	8.7 ± 4.3	2.5–19.4	**0.017**
Resistin (pg/ml)	942 ± 273	429–1416	999 ± 428	258–2558	0.664

Statistically significant values are given in bold

## Discussion

The rationale for our investigation was based upon numerous former studies indicating that glucocorticoids influence adipokine synthesis. Glucocorticoids stimulate leptin production in cultured rat and human adipocytes [31, 32] and may enhance insulin-induced leptin release in vitro and in vivo [32, 33]. In humans, most studies support a positive correlation between serum cortisol and leptin concentrations, as demonstrated in our control cohort [34]. Subjects with Cushing disease display consistently elevated leptin levels, which tend to gradually decrease after treatment [35, 36]. High dose, synthetic steroids, dexamethasone and prednisone, produce similar effect of leptin rise [33, 34, 37]. Accordingly, inhibition of adrenal cortisol synthesis with metyrapone induces a significant decrease in leptin secretion [38]. Nevertheless, a study assessing various 24 h HC infusion regimens revealed that short-term changes in circulating cortisol, which remained within the physiological range, did not affect leptin levels [39]. This would be in agreement with our finding that serum leptin in PAI appeared similar to healthy controls. In contrast to leptin, previous investigations of the relationship between adiponectin and glucocorticoids provided inconsistent results. In vitro studies indicate that glucocorticoids inhibit adiponectin secretion in humans and mice [40, 41]. In vivo analyses demonstrated positive as well as negative correlation between serum adiponectin and cortisol, which has not been elucidated even when free cortisol index or glucocorticoid bioactivity was considered [37, 42–44]. Diminished adiponectin levels were found in Cushing’s syndrome, although successful treatment not always leads to rise in adiponectinemia [35, 45]. In our study adiponectin levels did not differ between healthy and PAI individuals, suggesting that conventional steroid replacement does not affect adiponectin secretion. In line, daily HC dose, serum cortisol level, nor UFC excretion were correlated with adiponectin in PAI patients. Finally, according to the results of in vitro experiments, a positive relationship between resistin and cortisol could be presumed, corresponding to the supposition that resistin was involved in glucocorticoid-induced insulin resistance [46]. Elevated resistin levels were found in patients with Cushing’s syndrome; however, a curative surgery did not influence resistin concentration despite a fall in UFC and improved insulin sensitivity [35]. Increased serum resistin was also reported in subjects with adrenal incidentalomas displaying subclinical hypercortisolaemia [47]. However, short-term dexamethasone administration, which enhanced insulin resistance, did not alter circulating resistin levels in obese subjects [48]. In our study no correlation was detected between serum cortisol and resistin, therefore observed decrease in circulating resistin in PAI could not be explained by steroid deficiency.

Conventional oral glucocorticoid substitution implies intervals of supra-physiological cortisol levels hence it always raises concerns about possible adverse effects of long-term over-treatment [1–3]. Although far from perfect, this therapy does not seem to induce changes in circulating adipokines, which might be indicative of steroid excess. In our study leptin and adiponectin levels remained similar to healthy controls, matched for age, gender and BMI. Moreover, healthy and affected subjects presented no differences in fasting glucose and cholesterol profiles, both features involved in cardiometabolic risk. Mean (±SD) HOMA-IR in PAI patients (1.9 ± 1.7) does not support common insulin resistance, however, relatively high standard deviation value indicates that some of these subjects may exhibit impaired insulin sensitivity. Unfortunately, there is a great variability in the HOMA-IR thresholds in different populations therefore lack of data from our control cohort impairs accurate interpretation of these findings [49].

Recent reports from Sweden suggest increased cardiovascular morbidity and mortality in primary adrenal failure due to ischemic heart disease and cerebrovascular stroke [7, 8, 50]. Their authors postulate plausible role of inadequate steroid replacement. Glucocorticoids, which are known for their impact on cardiovascular system, may promote hypertension, visceral obesity, hyperglycemia and dyslipidemia [51]. Indeed, higher frequencies of these cardiovascular risk factors were demonstrated in PAI [3, 9, 10]. However, mean glycemic and lipid parameters were similar in Italian patients and controls [9]. A comparison between South African and Swedish PAI cohorts revealed significant population differences, with the Scandinavian results comprised within the reference range [52]. Moreover, lipid profiles evaluated in a large cohort of British patients on the occasion of a DHEA trial also appeared normal [53]. Unfortunately, the number of clinical studies addressing cardiovascular risk under glucocorticoid substitution remains limited. Swedish data, which support increased incidence of cardiovascular deaths in PAI, were derived from the national hospital and death registers [7, 8]. Health registers, even very meticulous, may be biased by variable local criteria of diagnosis and administrative reporting errors. Therefore, designed clinical studies seem a more reliable source of information. In a retrospective Norwegian study comprising the entire PAI population, cardiovascular disease appeared the most common cause of mortality, accounting for 39 % of deaths, but this value remained comparable to the general population [6]. On the whole, at present there is no definitive proof of increased cardiovascular morbidity and mortality among patients with PAI. Furthermore, our analysis of their serum adipokine profile did not reveal patterns typical for cardiometabolic disease and no correlation between circulating cortisol or HC dose and adipokine level was found. However, large prospective studies are still warranted to clearly establish if subjects receiving standard glucocorticoid substitution are at exacerbated cardiometabolic risk.

Correlations between circulating adipokines and anthropometric parameters in patients with PAI in our study were in keeping with previous findings from different lean and obese cohorts. Leptin levels appeared positively correlated with BMI and fat proportion, whereas an inverse relationship was confirmed for adiponectin and weight in PAI and healthy subjects [14, 18, 39, 45, 54]. HDL cholesterol emerged as a significant positive predictor of adiponectin level in both groups [14], which is in line with a favorable cardiometabolic profile associated with higher adiponectin concentration [45, 54, 55]. Finally, insulin resistance marker, HOMA-IR, revealed a positive correlation with leptin and a negative correlation with adiponectin level, although the latter disappeared in multiple regression analysis. Both correlations were formerly reported in several populations [39, 42, 45, 54, 55]. Leptinemia was even suggested to be a more robust surrogate marker of insulin sensitivity than fasting insulin [56]. Additionally, our analysis supports the relationship between resistin level and insulin resistance, even though this correlation was borderline and studied only in PAI cohort. Former data from humans remain elusive; however, some studies in healthy lean adults confirm resistin correlation with fasting insulin and HOMA-IR [39, 54].

Interestingly, both leptin and adiponectin revealed negatively correlated with DHEA-S levels in our PAI cohort. Similar relationship between leptin and DHEA-S was reported in hypoadrenal women receiving DHEA replacement, despite lack of change in BMI and body composition over the 4 months treatment period [57]. Moreover, leptin-lowering effect of androgen substitution was reported in hypogonadal men [58]. Inverse adiponectin correlation with adrenal androgen was previously described in congenital adrenal hyperplasia and polycystic ovaries syndrome [59, 60]. In our study, these correlations were lost in multiple regression analysis but prompted a post hoc analysis comparing adipokine levels in patients receiving DHEA substitution and those who were not under adrenal androgen replacement. Accordingly, leptin and adiponectin appeared significantly higher in non-substituted subjects (Table 2). These observations are in line with in vitro data, which confirm suppressive effect of androgens on leptin and adiponectin synthesis [18, 21]. In vivo, lower androgen levels may contribute to sexual dimorphism of circulating adipokine levels, which was also demonstrated in our healthy and affected cohorts [39, 54, 55, 61]. These results add up to the current discussion concerning metabolic benefits of the DHEA replacement in adrenal failure, which remain controversial despite several randomized trials [53, 62, 63].

To summarize, our study did not provide evidence of an adverse adipokine profile in patients with PAI under conventional glucocorticoid replacement. The adipokine levels in treated PAI individuals do not differ from the general population, and they follow similar correlations to those reported in healthy subjects. DHEA replacement may decrease serum leptin and adiponectin levels, but clinical relevance of this finding remains to be elucidated. Further studies are required to substantiate the epidemiological observations of increased cardiometabolic morbidity and mortality in PAI and to explore their pathomechanisms.
